# The role of MedDRA in global pharmacovigilance and public health during COVID-19: pandemic preparedness and response

**DOI:** 10.3389/fdsfr.2025.1607642

**Published:** 2025-11-25

**Authors:** Brian O’Hare, Heba Ibrahim, Patrick Revelle

**Affiliations:** 1 MedDRA Maintenance and Support Services Organization, Herndon, VA, United States; 2 MedDRA Maintenance and Support Services Organization, Cairo, Egypt

**Keywords:** MedDRA, ICH terminology, pandemic response, medical coding, safety analysis, standardised MedDRA queries (SMQs), system organ class, COVID-19 vaccine

## Abstract

The COVID-19 pandemic has had a profound impact on global public health, infecting over 770 million individuals and resulting in more than 6.9 million deaths worldwide. In response, the rapid development of novel vaccines became critical to controlling the spread of the virus and safeguarding public health. The accelerated rollout of these vaccines required an expedited evaluation of their quality, efficacy, and safety. This article highlights the role of the Medical Dictionary for Regulatory Activities Maintenance and Support Services Organization (MedDRA MSSO) and the International Council for Harmonisation of Technical Requirements for Pharmaceuticals for Human Use (ICH), the terminology owner, in addressing the pandemic’s challenges. The authors discuss MSSO’s swift adaptation of its standard procedures to the pandemic’s needs to develop and maintain MedDRA COVID-19 terms and tools. Through close collaboration with the ICH and expert working groups, the MSSO facilitated the development and timely updating of MedDRA terms and tools tailored to COVID-19, supporting global efforts in diagnosis, prognosis, and novel vaccine safety monitoring. The integration of these MedDRA terms and tools has been crucial in the timely reporting, coding, and analysis of adverse events following immunization (AEFIs), thereby facilitating public health responses worldwide and enabling the ongoing evaluation and safety assurance of COVID-19 vaccines.

## 1 Introduction

The costs of the COVID-19 (SARS-CoV-2) pandemic to public health have been significant worldwide. COVID-19 has affected the health of more than 770 million people, with more than 6.9 million deaths reported worldwide, in addition to devastating direct and indirect health outcomes (Msemburi et al., 2022). This pandemic was shortly followed by the introduction of innovative COVID-19 vaccine products. These novel vaccines have required expedited procedures to enable assessment of their quality, efficacy, and safety, and ultimately protect public health.

From the beginning, and in response to the pandemic, the International Council for Harmonisation of Technical Requirements for Pharmaceuticals for Human Use (ICH) (International Council for Harmonisation of Technical Requirements for Pharmaceuticals for Human Use, 1999), the Medical Dictionary for Regulatory Activities (MedDRA) Maintenance and Support Services Organization (MSSO) (MedDRA Mainte nance and Support Services Organization, 1999), and ICH expert working groups worked together to develop a rapid and unique response.

In this article, the authors outline the MSSO’s standard approaches to developing and maintaining MedDRA terminology and then explain its role in addressing global public health challenges posed by COVID-19. The authors describe the effective response of ICH and MSSO to the pandemic by accelerating the development and maintenance of new MedDRA terms and tools for COVID-19. These exceptional activities have played a pivotal global role in diagnosis, prognosis, indication, and prevention, as well as monitoring the safety of novel COVID-19 vaccines by enabling the timely reporting, coding, and analysis of adverse events following immunization (AEFIs).

## 2 MedDRA history and governance

MedDRA is the international medical terminology developed under the auspices of the ICH (International Council for Harmonisation of Technical Requirements for Pharmaceuticals for Human Use, 1999). A dedicated MedDRA Maintenance and Support Services Organization (MedDRA MSSO) was established to support its development and maintenance. MSSO’s first release of MedDRA (version 2.1) was in March 1999. When the European Medicines Agency (EMA), the Japanese Ministry of Health, Labour and Welfare (MHLW), and US Food and Drug Administration (US FDA) adopted MedDRA as a standard, MedDRA started on the path to its current role as an international standard. The ICH MedDRA Management Committee (MedDRA MC) is responsible for the direction of MedDRA and overseeing all the activities of MSSO which is charged with the maintenance, development, and distribution of MedDRA.

MedDRA is maintained in English and has been translated and maintained in several additional languages (MedDRA Maintenance and Support Services Organization, 2025b). MedDRA is available as a free subscription to all regulators worldwide, while paid subscriptions are on a sliding scale linked to the annual turnover of commercial organizations. Academics and healthcare providers can also access MedDRA at no cost.

To support the efficient use of MedDRA, the ICH M1 Points to Consider Working Group (M1 PtC WG) updates with each MedDRA release two PtC documents on MedDRA Term Selection and MedDRA Data Retrieval and Presentation (MedDRA Maintenance and Support Services Organization, 2025a). The fully translated version of the PtC documentation is available in six languages. While the condensed version is available in all other MedDRA translations (MedDRA Maintenance and Support Servi ces Organization, 2025b). In addition, since 2017, the scope of the M1 PtC WG has been expanded to include the maintenance of a companion document to the PtC documents which provides more detailed guidance, examples, and questions and answers on topics of regulatory importance (e.g., data quality, medication errors, and product quality issues) (ICH M1 PtC Working Group, 1999).

## 3 Scope and structure of MedDRA

MedDRA is a rich and highly specific standardized medical terminology for regulators and regulated biopharmaceutical industry to facilitate sharing of regulatory information internationally for medicinal products used by humans. MedDRA is used for the registration, documentation and safety monitoring of medical products both before and after a product has been authorized for sale. Products covered by the scope of MedDRA include pharmaceuticals, biologics, vaccines and drug-device combination products (Harrison and Mozzicato, 2009).

The scope of MedDRA includes all phases of the development of medicinal products for human use, excluding animal toxicology. This encompasses medical, health-related, and regulatory concepts pertaining to such products. The terminology also addresses the health effects and malfunction of medical devices. Furthermore, the terminology may support other types of products that are regulated in at least one region (e.g., food or cosmetics).

**FIGURE 1:**
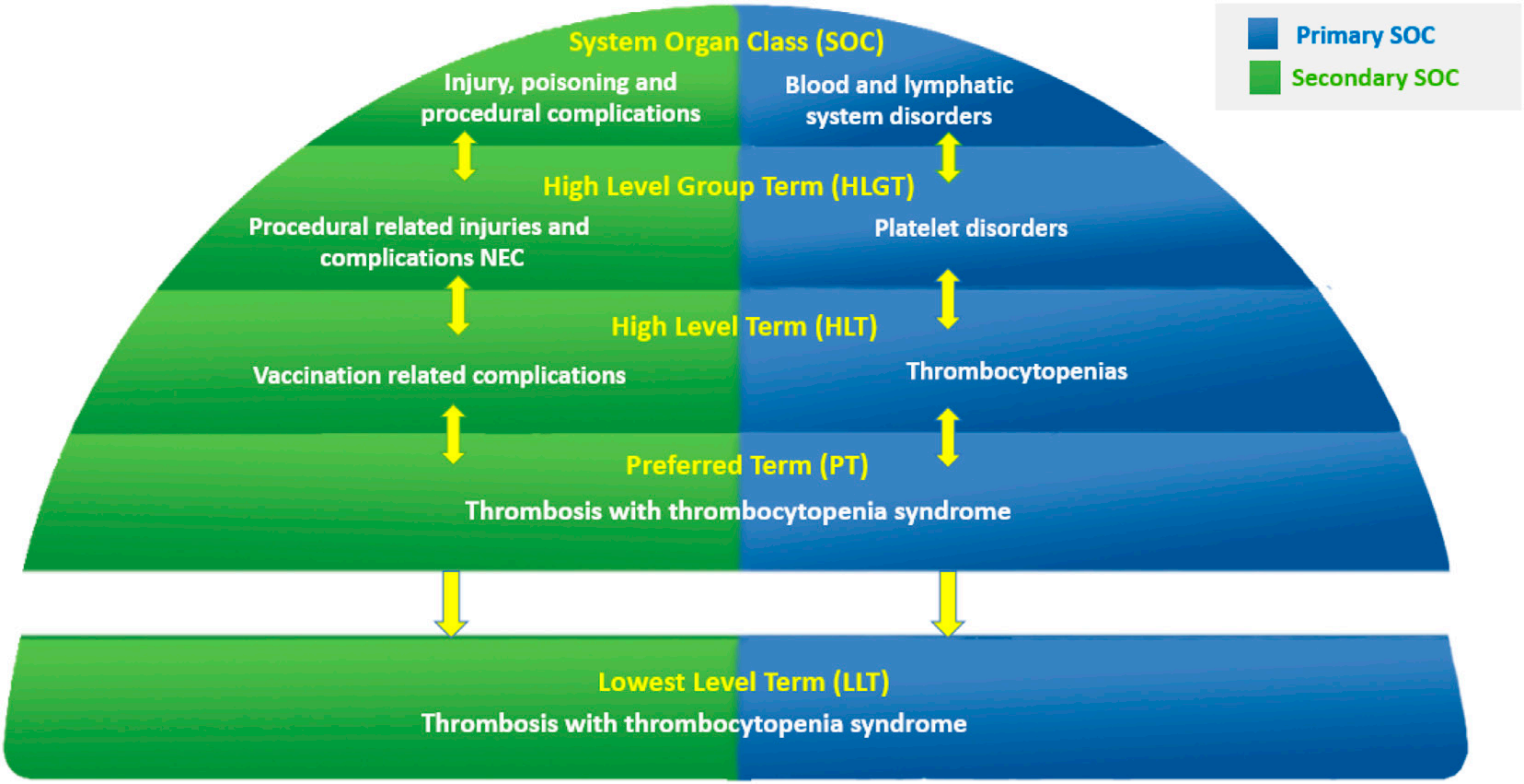
Example of MedDRA five-level multiaxial hierarchy.

MedDRA is structured as a five-level hierarchy (Figure 1), ranging from very specific to more general terms: Lowest Level Terms (LLTs), Preferred Terms (PTs), High Level Terms (HLTs), High Level Group Terms (HLGTs), and System Organ Classes (SOCs). It is grouped into 27 SOCs that represent parallel axes (Bousquet et al., 2014). This characteristic, known as “multiaxiality,” allows a term to be represented in more than one SOC and to be grouped by different classifications, enabling retrieval and presentation via multiple data sets. Multiaxial links of terms are pre-assigned, allowing automatic assignment of grouping terms higher in the hierarchy and ensuring comprehensive and consistent data retrieval, regardless of which SOC is selected at retrieval. Each MedDRA PT is assigned a primary hierarchy and, in some cases, secondary hierarchies (Brown et al., 1999). MedDRA also includes “Standardised MedDRA Queries (SMQs)”, which are groupings of MedDRA terms at the PT level that relate to a defined medical condition or area of interest (Brown et al., 1999). SMQs are intended to aid in the identification and retrieval of potentially relevant individual case safety reports (Chang et al., 2017; Hartford et al., 2006; Brown, 2004). The included terms may relate to signs, symptoms, diagnoses, syndromes, physical findings, laboratory and other physiologic test data, etc. The only LLTs represented in an SMQ are those that link to a PT used in the SMQ; all others are excluded.

## 4 Role of MedDRA in pharmacovigilance

MedDRA integration is an indispensable component of pharmacovigilance systems. It ensures data standardization, regulatory compliance, and the ability to effectively detect and assess safety signals (Harrison and Mozzicato, 2009; Chang et al., 2017; Hartford et al., 2006; Brown, 2004; Mozzicato, 2007). By supporting the entire pharmacovigilance process, from data collection and coding to analysis and reporting, MedDRA plays a fundamental role in drug safety monitoring and risk assessment, ultimately contributing to safer pharmaceutical products for patients worldwide. Some key aspects of the MedDRA role in pharmacovigilance can be summarized as follows:

− Standardization: MedDRA ensures consistency in reporting adverse events, facilitating uniform communication among researchers, healthcare professionals, and regulatory authorities worldwide.
− Regulatory Compliance: Many regulatory authorities, such as the FDA and EMA, mandate the use of MedDRA for reporting adverse events and conducting pharmacovigilance activities.
− Data Consistency and Aggregation: MedDRA helps ensure consistent encoding of medical terms across different databases and systems, allowing for the aggregation and analysis of data from various sources.
− Signal Detection: Early detection of safety signals is facilitated by MedDRA, enabling timely risk mitigation.
− Data Analysis and Reporting: MedDRA enhances the ability to generate consistent, structured safety reports and perform robust data analyses key for informing regulatory decisions and stakeholder communications.
− Data Interoperability: MedDRA includes cross-referencing capabilities, promoting interoperability between various pharmacovigilance databases.
− Improved Data Quality: By reducing data errors and inconsistencies, MedDRA enhances the reliability of pharmacovigilance data and safety assessments.

Through these capabilities, MedDRA substantively supports the robustness of pharmacovigilance systems, ensuring timely signal detection, effective regulatory communication, and high-quality safety data management while minimizing redundancy.

## 5 MedDRA maintenance process

As outlined in Sections 2, 4, the MSSO, under the oversight of the ICH MedDRA MC, is responsible for the continued development and biannual releases of MedDRA to ensure alignment with evolving medical knowledge and pharmacovigilance needs (Harrison and Mozzicato, 2009). Releases occur on 1 March and 1 September in English, with translated versions in 23 supported languages following on 15 March and 15 September. MedDRA users can submit change requests (CRs) – such as adding new terms, modifying existing ones, or updating SMQs and translations–through an online MedDRA tool, called “WebCR” (MedDRA Maintenance and Support Services Organization, 2008). Each CR undergoes a structured review by the MSSO medical officers, including an international panel, to ensure compliance with MedDRA’s rules and conventions, medical accuracy, completeness, and internationally accepted medical standards. Approved changes are incorporated into the MedDRA data files following quality checks, and the updated user documentation made available for download via the MedDRA website (Harrison and Mozzicato, 2009).

## 6 ICH/MSSO pandemic response and preparedness to support novel COVID-19 vaccine safety monitoring efforts

### 6.1 Special MedDRA release 23.0 for COVID-19 disease outbreak

By March 2020, the World Health Organization (WHO) had declared COVID-19 a global health emergency (Cucinotta and Vanelli 2020). The pandemic induced an urgent need for a standardized approach to code, analyze, and share safety data related to the infection and its treatments.

MedDRA Version 23.0, released on 1 March 2020, did not yet contain terms related to COVID-19. On 10 April 2020, the ICH M1 PtC WG and the MSSO, with MedDRA MC approval, issued a special out-of-cycle update introducing coronavirus-related terminology (International Council for Harmonisation of Technical Requirements for Pharmaceuticals for Human Use, 2020a).

**FIGURE 2:**
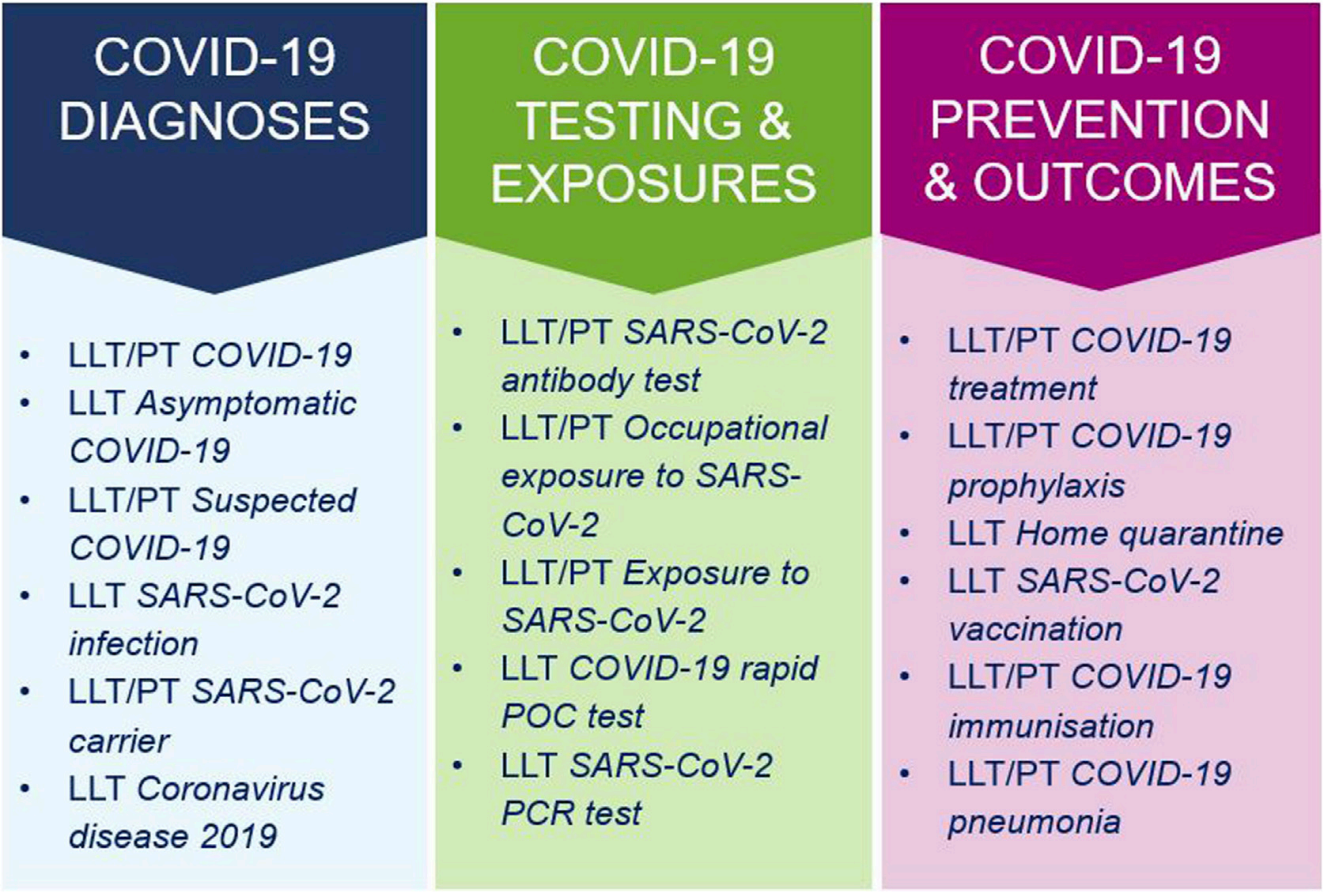
Categories and examples of COVID-19 terms in the out-of-cycle MedDRA release 23.0 (April 2020). LLT, Lowest Level Term; PT, Preferred Term.

On 19 April 2020, an out-of-cycle release of MedDRA version 23.0 (see Figure 2) introduced 68 new COVID-19 terms along with associated terminology revisions (International Council for Harmonisation of Technical Requirements for Pharmaceuticals for Human Use, 2020b; International Council for Harmonisation of Technical Requirements for Pharmaceuticals for Human Use, 2020c). These additions included the new HLT *Coronavirus infections* in SOC *Infections and infestations*, with most diagnostic terms located in SOC *Investigations*, and treatment/exposure concepts placed in SOC *Surgical and medical procedures* and SOC *Injury, poisoning and procedural complications*. These terms supported harmonized reporting and analysis of COVID-19 cases across regulatory and scientific settings, addressing both pre- and post-marketing safety data.

To support adoption, the MSSO communicated extensively with users and conducted two webinars on 17 and 20 April 2020, reaching over 1,100 participants. These sessions covered the background and scope of the update, global implementation timelines, and user Q&A.

Ongoing updates have continued in subsequent releases. MedDRA Version 23.1 added over 60 new COVID-19 LLTs/PTs. Version 24.0 introduced additional terms for congenital SARS-CoV-2 infection and post-acute COVID-19 syndrome. A comprehensive list is maintained on the COVID-19 page of the MedDRA website (MedDRA Maintenance and Support Services Organization, 2020).

### 6.2 Expediting development of standardized MedDRA query for COVID-19

**FIGURE 3:**
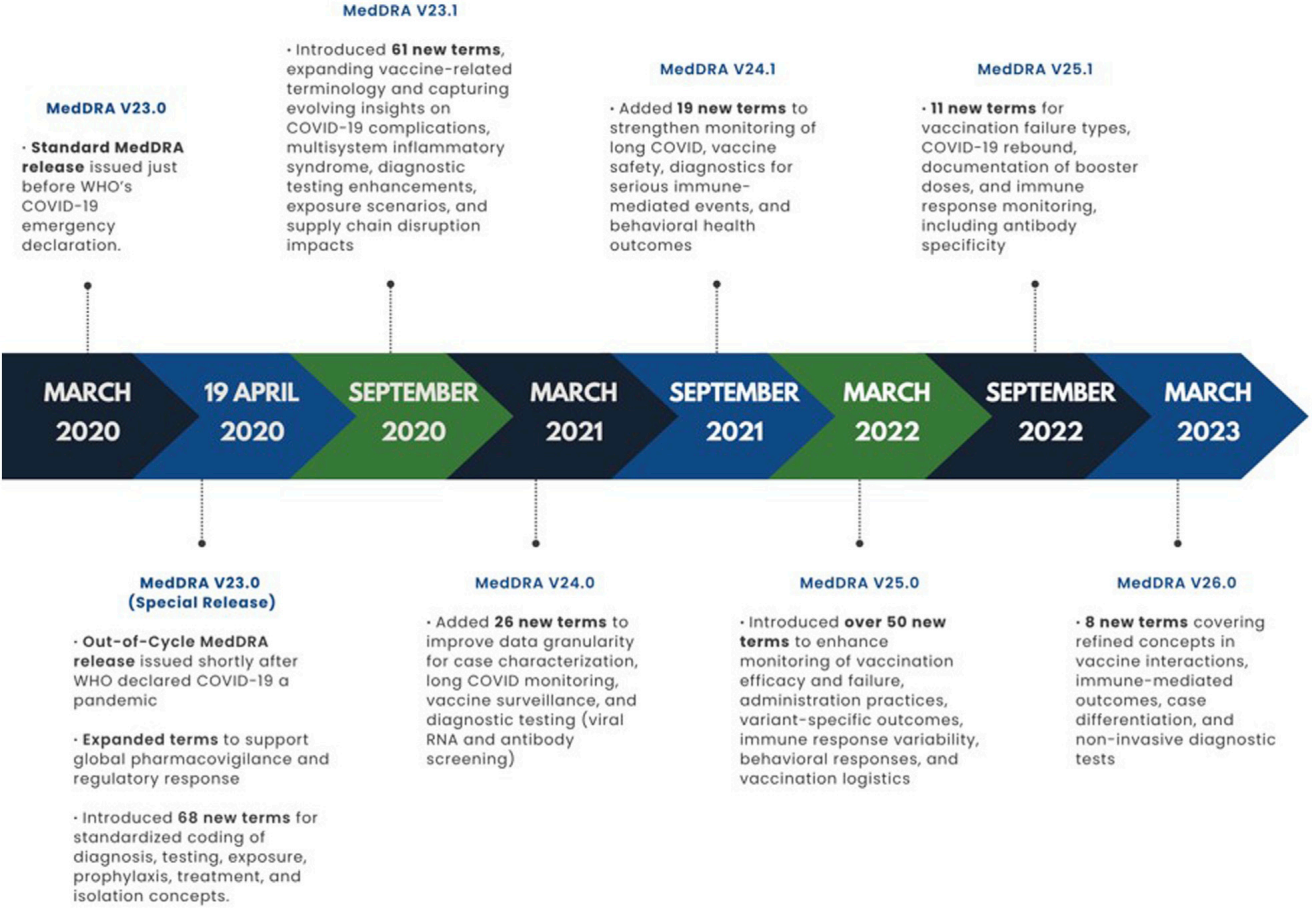
Development of COVID-19 terminology across MedDRA releases in the pandemic period (2020–2023).

To meet the urgent pharmacovigilance needs of regulators and the pharmaceutical industry during the pandemic, MSSO added a new “Standardised MedDRA Queries (SMQs)” for COVID-19 to MedDRA Version 23.1, released in September 2020 (Figure 3).

The *COVID-19 (SMQ)* was developed by the MSSO and an international group of experts in an expedited manner, based on medical judgment (International Council for Harmonisation of Technical Requirements for Pharmaceuticals for Human Use, 2020c). This SMQ could be utilized in a variety of scenarios, not only in adverse event data fields, but also in other relevant data fields such as those for medical history, indications, laboratory tests, etc. It can be used to identify and record cases of SARS-CoV-2 infection/COVID-19, and to capture information about other aspects of the pandemic, including testing and exposures. In the context of clinical trials, for example, the SMQ can aid in capturing COVID-19 related information as adverse events, inclusion criteria, indications for use, and reasons for disruption of trial conduct. Applications in pharmacovigilance can also include recording instances of off-label use of medical products for treatment or prevention of COVID-19. The SMQ can also be applied in databases to capture and analyze population level data about frequencies of cases, exposures, testing, and identification of populations at risk.

In anticipation of the rollout of SARS-CoV-2 vaccines and the global focus on monitoring the efficacy and safety of these vaccines, the MSSO also proactively reviewed the vaccine-related terms in MedDRA and sought solicited feedback from an international group of regulatory and industry users on the MSSO’s proposals for new terms for release 24.0. As an outcome of this review, several new terms and changes related to vaccine concepts including adverse events following immunization (AEFIs) were implemented in MedDRA V24.0 (March 2021) (Kralova et al., 2023; Adjaï et al., 2024; Dong et al., 2024; Rudolph et al., 2022).

The timeline of MedDRA releases during the COVID-19 pandemic reflects the rapid, stepwise, and iterative approach taken to address emerging clinical and regulatory needs. To visually illustrate this, Figure 3 presents a chronological summary of key versions from 23.0 (March 2020) through 26.0 (March 2023), mapping each release to its main COVID-19 terminology updates from early diagnostic and treatment concepts to vaccine safety monitoring, long COVID surveillance, and variant-specific outcome assessment.

### 6.3 MSSO and WHO/UMC collaboration on COVID-19 in VigiFlow vaccine surveillance

The first step in ensuring the safe administration of vaccine products is effective spontaneous reporting of AEFIs. To support the needs of AEFI surveillance programs in terms of descriptive epidemiology, and surveillance performance, the World Health Organization (WHO), in collaboration with the Uppsala Monitoring Centre (UMC) launched a digitalized WHO AEFI reporting form in the VigiFlow interface in January 2021 (Uppsala Monitoring Centre, 2021). The MSSO has collaborated with UMC to map a predefined list of most frequent AEFI terms to MedDRA (e.g., LLT/PT *Irritability postvaccinal*). This, therefore, provided VigiFlow users with multiple options for reporting AEFIs either by entering free text, selecting appropriate terms from the full MedDRA dropdown list, or selecting from the predefined list of AEFIs mapped to MedDRA.

### 6.4 Global MedDRA uptake during the COVID-19 pandemic

The outbreak of COVID-19 in early 2020, followed by the global pandemic, changed everything. By March 2020 and into early 2023, face-to-face training came to a complete halt as a result of travel restrictions and stay-at-home policies in many countries. The health crisis highlighted the importance of information collection and reporting, which provided more needed data for evidence-based decision-making by regulators and other organizations. Training was needed more than ever. To continue to provide training opportunities to MedDRA users during the pandemic, the MSSO quickly transitioned from in-person training to 100% virtual webinars. The MSSO training team increased the number of webinars to fill the void of face-to-face classes. Several enhancements to webinars were implemented to maximize interactivity between trainers and attendees in the virtual training environment and to accommodate global time zones. These included interactive polling in exercises, Q&A sessions, and live demonstrations during coding and analysis webinars. The polling tool offered multiple-choice, free-text entry, and image-click options, allowing attendees to actively participate in class.

## 7 Impact of pandemic on MedDRA terminology and global pharmacovigilance

### 7.1 Capturing individual case safety reports for COVID-19 treatments

Given the global scale of the pandemic, it has been crucial to promptly report information related to COVID-19 disease and suspected side effects from medications used to treat confirmed or suspected COVID-19 infection, as well as how treatments for long-term, pre-existing conditions are affected by the virus or its therapies, thereby supporting the analysis of incoming data.

The selection of precise COVID-19 term(s) when coding individual case safety reports (ICSRs) should not be limited to indications for use or reaction(s)/adverse event(s), but should also include, for example, aggravation/exacerbation of COVID-19 infection, relevant medical history and comorbid conditions, diagnostic test results, autopsy findings, etc.

Therefore, in line with the ICH E2B guidelines, the MedDRA term selection PtC document, and the special MedDRA Release 23.0 for COVID-19 terms (International Council for Harmonisation of Technical Requirements for Pharmaceuticals for Human Use, 2020a; World Health Organization, 2020), several global and regional recommendations have been issued to capture and code ICSRs related to COVID-19 treatments, thus supporting better understanding of efficacy and safety of those treatments (Uppsala Monitoring Centre, 2021; World Health Organization, 2020). This information may be submitted as an ICSR, discussed in the periodic safety update report, and/or addressed in the product risk management plan.

### 7.2 Standardized MedDRA query for safety monitoring of COVID-19 treatments and vaccines

The global response to the COVID-19 pandemic placed unprecedented demands on pharmacovigilance systems to rapidly identify, assess, and monitor the safety and effectiveness of emerging treatments and vaccines. This urgency elevated the role of SMQs, which became essential tools for targeted case retrieval and signal detection in the context of COVID-19 (Baden et al., 2021; di Mauro et al., 2020; Lee et al., 2023).

While the initial *COVID-19 (SMQ)*, released in MedDRA Version 23.1 in September 2020, laid the foundation for improved case identification, its effective use required specific implementation guidance provided by the MSSO. Users were advised to apply the SMQ not only to adverse event data, but also to other relevant fields such as indications, medical history, and laboratory test results. Special attention was also given to selecting appropriate date ranges, particularly for cases reported after late 2019, when the novel coronavirus first emerged. Additionally, the option to use narrow or broad search scopes supported a tailored balance between sensitivity and specificity in case retrieval. This flexible and context-sensitive application of the SMQ reflected the evolving needs of global pharmacovigilance during the pandemic.

Other existing SMQs can be used individually or in combination with *COVID-19 (SMQ)* to build a more comprehensive search strategy for the various clinical manifestations of the COVID-19 and associated adverse drug events or AEFIs (Caplanusi et al., 2024). For example, the *Embolic and thrombotic events (SMQ)* has been used to retrieve thromboembolic cases, detect, and assess AEFI signals of thrombosis with thrombocytopenia syndrome following vaccination with novel adenoviral vector-based COVID-19 vaccines (Woo et al., 2022; Procter et al., 2023).

## 8 Discussion

The updated release of MedDRA Version 23.0 was well received by the global MedDRA user community. In response to the urgent need for rapid, standardized terminology during the COVID-19 pandemic, the ICH MedDRA MC, in collaboration with the MSSO, acted swiftly to support vaccine manufacturers and regulators. New MedDRA terms were introduced to capture key concepts related to COVID-19 vaccine safety and efficacy, enabling consistent data submission and independent analysis across regulatory authorities. The ability to deliver an out-of-cycle release - while maintaining the integrity of MedDRA’s quality standards - was a significant accomplishment.

A key lesson learned from this effort was the critical importance of organizational preparedness. In response, MSSO has expanded its internal standard operating procedures and work instructions to accommodate similar public health urgent scenarios in the future. The COVID-19 pandemic reinforced affirmed MedDRA’s role as a reliable and adaptable pharmacovigilance tool, of supporting timely responses to global health emergencies.

These MedDRA adaptations, though driven by the COVID-19 pandemic, demonstrate broader utility for future public health challenges, such as emerging infectious diseases, pandemic preparedness, and vaccine safety monitoring. The expanded and continuously maintained terminology can support timely and standardized data capture across diverse clinical and regulatory contexts.

Nonetheless, the key challenge in this process was determining how and which elements of the standard 6-month MedDRA maintenance cycle could be effectively condensed into just a few days without compromising the stringent quality controls applied to all MedDRA releases. While the flexibility of existing tools and the expertise of the technical team were critical in enabling this accelerated response, some aspects of the rapid implementation did not proceed as smoothly. Specifically, coordination across regions presented difficulties due to differences in regulatory timelines, local approval processes, and varying levels of readiness to integrate new terminology. These discrepancies occasionally led to delays in the uptake of updated terms by national authorities and users, reflecting broader challenges noted in cross-regional regulatory collaboration and real-world evidence integration (Beck et al., 2025).

Additionally, effective communication between the ICH MedDRA MC, MSSO, and MedDRA users supported agile and timely decision-making. However, the rapid onset of the pandemic underscored the importance of strengthening outreach efforts, particularly in regions with varying access to regular communication channels. From the MSSO’s perspective, a key takeaway from this experience was the importance of expanding the use of digital platforms (e.g., WhatsApp, Facebook, LinkedIn, WeChat) to support timely and inclusive information sharing during other potential disruptions.

## Data Availability

The original contributions presented in the study are included in the article/supplementary material, further inquiries can be directed to the corresponding author.
